# Technical and academic scenarios for healthcare technology evaluations: Cochrane Library impact factor assessment of 4.65!

**DOI:** 10.1590/S1516-31802008000400001

**Published:** 2008-07-03

- Starting with this issue, following an initiative from the Scientific Department of São Paulo Medical Association (Associação Paulista de Medicina, APM) and with support from the National Council for Scientific and Technological Development (Conselho Nacional de Desenvolvimento Científico e Tecnológico, CNPq), for inclusion in the ISI database (Institute for Scientific Information), the São Paulo Medical Journal will be published with the subtitle “Evidence for Health Care”. This change is being implemented because the culture of Evidence-Based Medicine is becoming increasingly consolidated in Brazil, thanks to initiatives from APM through the São Paulo Medical Journal, the journal Diagnóstico & Tratamento, the Scientific Department and the Board of Directors, and also through the television program “APM on the TV: Evidence-Based Medicine”, which is now back on the air, supported by the whole APM Board. Furthermore, we already have scientific production of quality and quantity to supply the initial editorial needs.
  The Journal will keep the same characteristics that have provided it with all the requisites for recognition of its international standards. It will be expanded to enable publication of articles that are deemed to provide evidence for clinical practice and implementation, such as systematic reviews following the Cochrane standards, summaries of Cochrane reviews, clinical management and evidence-based technological assessments.
  Authors of papers in these fields are more than welcome to submit their work for consideration. At the same time, following the practice of the Cochrane Collaboration to disseminate the best evidence, articles of national interest may be reproduced in the São Paulo Medical Journal, provided that this is authorized by the Cochrane Library's editors and the authors concerned, and this will be made clear in the Journal. In parallel with this, we will comply with all the requirements for the São Paulo Medical Journal to become indexed in the Web of Science (ISI).
- In chapters on healthcare technology evaluations in two recently published books that are opportune and otherwise recommendable, the opinion given is that it is almost impossible to accomplish such evaluations in Brazil, because of the lack of human resources and because of their complexity.^1,2^
  We completely disagree with this. This would signify total surrender in the face of a vital challenge for Brazilian Medicine and would constitute a declaration of little faith in the quantity and quality of Brazilian healthcare professionals, including those with master's and doctoral degrees who have already formally qualified, and including those under the supervision of one of the authors cited. It also ignores all the good work developed by the Brazilian Cochrane Center, APM, the Brazilian Medical Association (Associação Médica Brasileira, AMB), the Ministry of Health, the National Agency for Sanitary Surveillance (Agência Nacional de Vigilância Sanitária, ANVISA) etc.
  Nonetheless, this controversy is good and we are not going to shy away from it. We take the view that everyone will have to agree that it only makes sense to proceed with a technological assessment on a new diagnostic, therapeutic or preventive method, if there is **evidence** regarding its efficacy or effectiveness, at least. This is even clearer if there is no proof of its efficiency and safety, considering that in these evaluations we are generally investigating the advantages of the new product in relation to non-intervention or other options. Therefore, if there is no reasonable evidence that, for example, the treatment has better performance than non-intervention in the real world, there is no justification to continue to evaluate it technologically, with regard to its economic, management and safety characteristics, etc. Good-quality randomized clinical trials are a fundamental step in this process.
  For instance, if a type of prosthesis or medication is clearly less effective than and not as safe as a similar product, there is no economic evidence that can recommend it, no matter how financially or managerially advantageous the item may be, since safety is essential. Moreover, without safety, there is little room to continue with a more pragmatic assessment. Nor does it make sense to continue with a technological assessment on products when clinical trials on these products have demonstrated that their effect is no better than placebo, for example.
  A study of ours that mapped out the evidence for first-level healthcare decisions (Cochrane systematic reviews)^3^ showed that for 50% of the treatments reviewed, not even a comparative clinical trial had been published, and that in less than 10% of the interventions was there sufficient certainty for further studies not to be justified. In other words, in 50% or more of the occasions, an assessment of the evidence relating to effectiveness, efficiency and safety would be enough to complete the process of technological assessment, which has the aim of achieving more immediate implementation. Hence, adequate mapping of the evidence is essential for the technological assessment of any procedure, and this will resolve a large proportion of the cases. We reemphasize that mapping out the evidence is mandatory for starting the process, and it may often finish the process. When there is evidence that an item is effective and safe, then indeed we can move on to evaluations of the economics, efficiency, management, ethics and so on.
- The impact factor of the Cochrane Library in the ISI database (see Annex) has been measured as 4.65. This is several times greater than the impact factor of the Brazilian medical journal that is most cited, the Brazilian Journal of Medical and Biological Research. Congratulations to the several hundred reviewers of the Brazilian Cochrane Collaboration and our thanks to everyone who collaborated in this historic undertaking.

## ANNEX

Dear all,

The 2007 impact factors (IFs) have now been published by Thomson ISI, and the Cochrane Database of Systematic Reviews has an IF of 4.654 and is ranked 14 out of 100 in the ISI category Medicine, General & Internal.

Congratulations to you all.

The 2007 IF is calculated on the total number of cites in 2007 to articles published in 2006 (2442 cites) and in 2005 (2798 cites) total = 5,240 divided by the total number of articles published in 2006 (575) and 2005 (551) total= 1126.

As noted in my previous email, we will be analyzing the data available from ISI to form future communications. In the meantime, should you have any questions please feel free to contact me.

Best wishes,
